# Seasonal dynamics of microbial communities and potential human pathogens in *Crassostrea hongkongensis* and ambient water in southern China

**DOI:** 10.1128/aem.01056-26

**Published:** 2026-09-02

**Authors:** Xiaoxia Liu, Xianyu Wang, Haipeng Zhu, Wenqi Chen, Xin Hong, Yi Wan, Jiaomei Huang, Chunsheng Liu

**Affiliations:** 1 School of Marine Biology and Fisheries, Hainan University 74629, Haikou, China; 2 Key Laboratory of Marine Microorganism Technique of Haikou, Haikou, China; 3 School of Breeding and Multiplication, Hainan University 74629 , Sanya, China; University of Delaware, Lewes, Delaware, USA

**Keywords:** *Crassostrea hongkongensis*, host-associated microbiota, foodborne pathogen, seasonal variation, *Vibrio*

## Abstract

Oysters are a popular raw seafood worldwide, yet their contamination with human pathogens poses substantial public health risks. However, the seasonal dynamics of host-associated microbiota and the environmental drivers of pathogen accumulation in *Crassostrea hongkongensis* remain poorly understood. Here, we conducted 16S rRNA amplicon sequencing to profile the gill and intestinal microbiota of *C. hongkongensis*, sampled quarterly over a 1-year period in Beihai and Zhanjiang, southern China. A pronounced divergence in oyster-associated bacterial communities was observed between the two locations in December. Key bacterial families, such as Lachnospiraceae and Muribaculaceae, showed significant temporal fluctuations in abundance, suggesting their potential role in maintaining bacterial community stability within oysters. These microbial shifts were significantly correlated with environmental parameters such as chlorophyll *a* and pH. In parallel, we used plate counting to quantify five foodborne pathogens and total viable counts. Vibrios were more frequently detected in oyster tissues than in ambient seawater. Pathogen-associated amplicon sequence variants exhibited a strong response to variations in pH and chlorophyll *a*, whereas the abundance of culturable *Vibrio vulnificus* was significantly negatively correlated with temperature and nitrate. The integration of high-throughput sequencing and culture-based methods provides comprehensive insights into the dynamics of pathogenic bacteria within oysters under natural mariculture settings. This study offers valuable ecological insights into the interactions within the oyster-associated microbial ecosystem, contributing to a deeper understanding of its dynamics and implications for public health.

**IMPORTANCE:**

Mariculture of *Crassostrea hongkongensis* serves as a critical interface between coastal ecology and public health, yet seasonal dynamics of its microbiome and core stabilizing taxa remain poorly understood in subtropical aquaculture. This study identified Acetobacteraceae, Lachnospiraceae, Prevotellaceae, and Muribaculaceae as key families associated with seasonal microbiome stability in *C. hongkongensis*, highlighting their potential contribution to community resilience. It further revealed that *Vibrio* species detection was negatively correlated with temperature. These findings advance our understanding of interactions between the oyster microbiome and the environment, and provide a theoretical basis for improving oyster health management and promoting sustainable aquaculture practices.

## INTRODUCTION

*Crassostrea hongkongensis* is salinity tolerant (15–30 ppt) (1) and can experience dramatic temperature fluctuations (10℃–40℃) (2). As well-known for its excellent meat quality, *C. hongkongensis* is one of the commercially significant farmed shellfish in southern China. It was principally distributed along the coastal waters of Guangdong and Guangxi, with an annual production of approximately 2 million tons (3). Additionally, it also plays a pivotal role in water filtration and maintenance of water quality (4). It has been reported that oysters may provide excellent ecological services in cleaning human-infectious agents from ambient water (5). However, pathogens may accumulate in their tissues, causing seafood-borne disease (6). The rising popularity of oysters has led to increased consumption, primarily raw-oyster consumption, which may result in outbreaks of human diseases, posing a global public health threat.

It is urgent to understand the background information of the aquatic environment and oyster ecology and the lifestyle of the microbial community, especially the occurrence and persistence of potential human pathogens in oysters. Recently, 16S rRNA gene amplicon sequencing has been used to determine the composition, diversity, and dynamics of oyster-associated microbiota. Gill-associated microbial communities in *Crassostrea gigas* were dominated by *Sphingomonas* and *Mycoplasma* (7), while the major members in oyster gut microbiota were Cyanobacteria, Proteobacteria, Tenericutes, and Actinobacteria (8). The distinction between oyster and seawater microbiota was also investigated, showing that microbial species richness was much higher in the seawater than in *C. gigas* of the Wadden Sea (9). Seasonal changes in oysters have been observed, with microbial concentration and diversity increasing in the warmer months and declining in colder months (10, 11). Microbial communities associated with water and *C. hongkongensis* were also reported before. The richness of culturable heterotrophic bacteria in water and the gut of *C. hongkongensis* peaked in August. It also showed that salinity had a more significant impact on seawater compared to temperature, while temperature had a more significant impact on oyster (12). However, the knowledge of ecology and function of microbial community in *C. hongkongensis* is still limited.

*Vibrio parahaemolyticus* and *Vibrio vulnificus* are indigenous to coastal environments; both can be concentrated in filter-feeding shellfish and have a negative impact on human health (13, 14). Pathogenic *V. parahaemolyticus* organisms in oyster and water samples were detected frequently, and the *V. parahaemolyticus* density has been shown to be positively correlated with water temperature (15–17). In addition, *Listeria monocytogenes*, fecal coliforms, and *Salmonella* spp. were also isolated from oysters (18, 19). Previous studies have also demonstrated that when *Mytilus edulis and Crassostrea gigas* were placed in seawater with *Escherichia coli*, the bioaccumulation of *E. coli* could persist for longer periods over 48 h (20). These findings indicated a necessity for specific monitoring of these pathogens.

Although Beihai and Zhanjiang are major harvesting areas for *C. hongkongensis*, the seasonal dynamics of oyster-associated microbial communities, opportunistic pathogen occurrence, and their relationships with environmental variables remain poorly understood. This is in contrast to the extensive research available for other major *Crassostrea* species. For instance, studies on *C. gigas* in temperate regions have established that its microbiota undergoes predictable successions primarily driven by thermal shifts (21, 22), while research on *Crassostrea virginica* has highlighted that their gut microbiota is co-regulated by host intrinsic factors (species specificity) and extrinsic factors (food and temperature) (23). To address this knowledge gap, this study systematically investigated the seasonal dynamics of microbial communities in seawater and in the gill and intestine of *C. hongkongensis*, with a focus on dominant bacterial taxa and potential human pathogenic populations. We hypothesize that microbial communities associated with oysters and surrounding seawater exhibit seasonal fluctuations, with oyster-associated microbiota displaying greater temporal stability than seawater microbial assemblages. In the marginal tropical region of subtropical aquaculture systems, temporal variations in microbial community structure are associated with environmental variables and may exhibit distinct regional signatures. By investigating seasonal microbial dynamics and their relationships with environmental factors, this study aims to provide ecological insights into oyster-associated microbiota and pathogen dynamics in subtropical mariculture environments.

## MATERIALS AND METHODS

### Sample collection, processing, and storage

**Fig 1:**
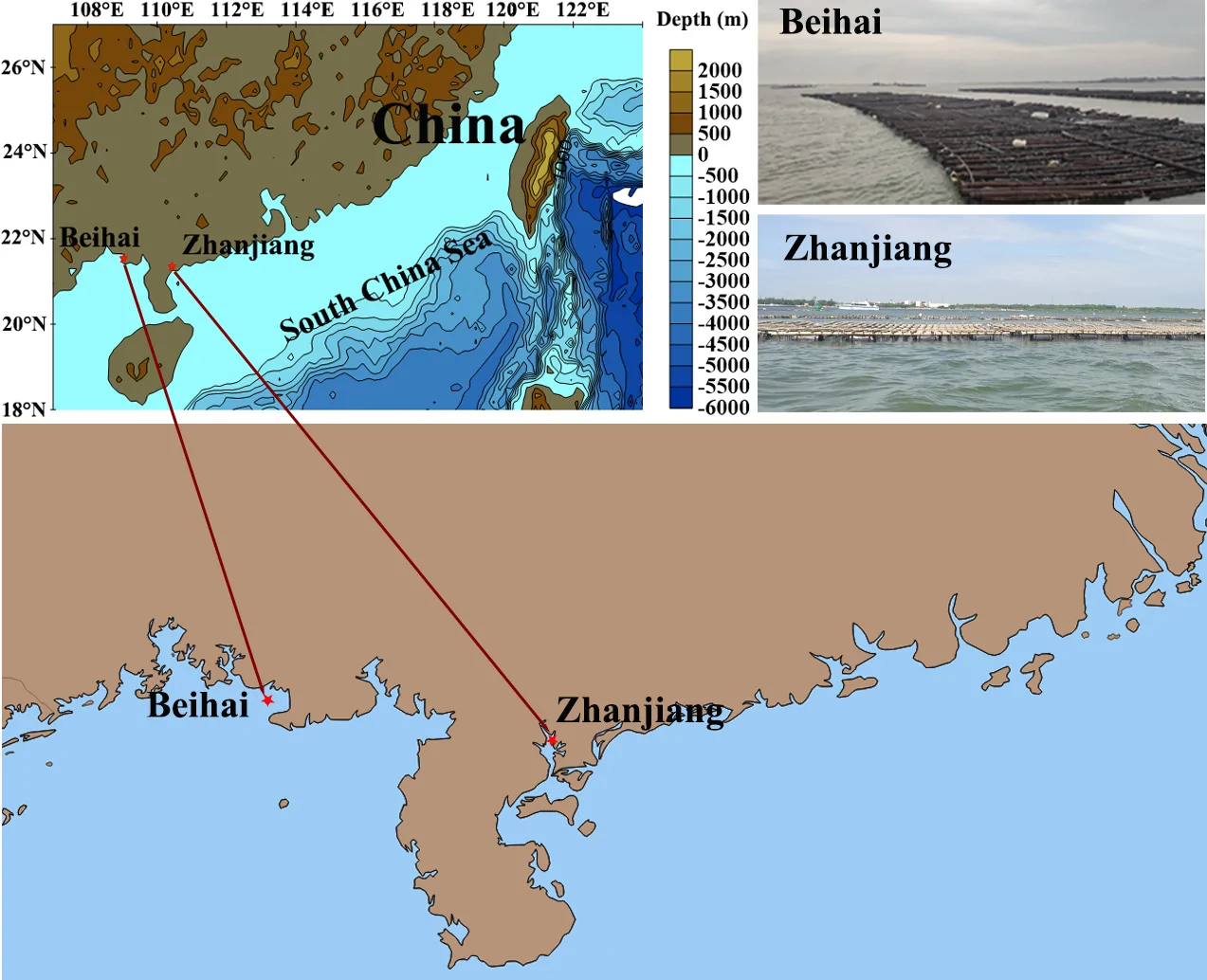
Oyster harvested locations in Beihai and Zhanjiang, China. Maps made with Natural Earth.

Adult *C. hongkongensis* from the same family were collected quarterly (June, September, and December 2023 and March 2024) at two locations in China (Fig. 1). One location was in Beihai, Guangxi Province (109°1′56.2944″ E, 21°32′30.5592″ N), and the other was in Zhanjiang, Guangdong Province (110°25′22.5588″ E, 21°20′9.5856″ N). Both locations were approved shellfish-growing areas with commercial harvesting. The detailed biological characteristics of the oysters collected at each site are summarized in Table 1. Oysters (30 individuals from each site at each time) were placed on ice, transported to the laboratory, and dissected. In addition, ambient water was collected from the water layer (1–3 m depth) where oysters were cultivated using a water sampler. For the analysis of waterborne 16S rRNA and culturable foodborne bacteria, water samples were transferred into 500 mL sterile bags. For the determination of ammonia, nitrate, nitrite, and phosphate, water samples were collected into 500 mL polyethylene bottles. All collected water samples were placed on ice immediately after collection and transported to the laboratory within 2 h for further processing.

**TABLE 1:** Biological characteristics of oysters at different sampled months^*a*^

Season	Beihai			Zhanjiang		
	Shell length (mm)	Body weight (g)	Ratio of soft tissue (%)	Shell length (mm)	Body weight (g)	Ratio of soft tissue (%)
June 2023	92.85 ± 18.44^a^	104.33 ± 11.18^b^	11.35 ± 1.89^b^	75.94 ± 7.07^a^	67.09 ± 17.46^c^	12.24 ± 2.04^b^
September 2023	99.39 ± 36.57^a^	113.74 ± 39.94^ab^	13.04 ± 3.31^b^	83.49 ± 24.03^a^	98.58 ± 26.76^bc^	15.60 ± 3.23^b^
December 2023	109.19 ± 18.65^a^	139.39 ± 30.48^ab^	20.36 ± 5.19^a^	92.15 ± 11.76^a^	141.68 ± 30.55^ab^	16.70 ± 3.33^ab^
March 2024	115.63 ± 13.30^a^	150.63 ± 26.80^a^	22.87 ± 6.31^a^	87.42 ± 9.89^a^	154.37 ± 39.12^a^	22.91 ± 5.56^a^

^*a*^ Data represent means ± SD (15 oysters were calculated in each time point and each site). Values in a column with different letters indicate significant differences among treatments (*P *< 0.05).

Water temperature, salinity, chlorophyll *a*, and pH were measured by a YSI Multi-parameter Water Quality Monitor. In addition, ammonia, nitrate, nitrite, and phosphate were analyzed according to the specification for marine monitoring in China (GB/T 12763-2007) (24). Briefly, water samples were filtered through an acetic acid membrane filter (0.45 μm pore size) and collected into glass bottles, which were then stored at −20°C until analysis. Concentrations of dissolved inorganic nutrients (ammonium, nitrite, nitrate, and phosphate) were determined using the segmented flow analysis method on a SEAL Analytical AA500 Auto-analyzer System.

### DNA extraction and amplicons sequencing

Each water sample (1 L) was filtered through 0.22 μm polypropylene, and five replicates were prepared for microbial community analysis at each site and time point. Oyster samples were dissected and retained gills and intestines. The tissues (gill and intestine, each approximately 0.2 g) of 15 oysters were separately collected. For each tissue type, samples from three oysters were combined to form a single composite sample, which was then used for downstream analysis (a brief experimental procedure is shown in Fig. S1). The tissues or water DNA were extracted following the instructions of the High Pure PCR Template Preparation Kit (Roche, Basel, Switzerland). The quality of DNA was measured with gel electrophoresis and a Qubit 3.0 fluorometer (Invitrogen, Carlsbad, CA, USA). The amplification of the 16S rRNA gene V3–V4 region was performed using the primers 338F (5′-ACTCCTACGGGAGGCAGCA-3′) and 806R (5′-GGACTACHVGGGTWTCTAAT-3′). The resulting amplified product was then used to construct a DNA library with the TruSeq Nano DNA LT kit (Illumina, San Diego, CA, USA) and sequenced on an Illumina NovaSeq platform (2 × 250 bp).

### Bioinformatics analysis of 16S rRNA gene amplicons

The bioinformatics analysis of the amplicon sequencing data followed the QIIME 2 (v2024.2.0) pipeline (25). Details are shown as below: low-quality base at the ends and chimera were removed, then denoised, and merged by DADA2 (26) (--p-trunc-len-f 220, --p-trunc-len-r 220, and --p-chimera-method consensus) to get amplicon sequence variants (ASVs); the taxonomy of ASVs was classified with classify-sklearn QIIME 2 Feature Classifier to search against Silva (v138) database (27) with default parameters. For ASVs subscribed to mitochondria, unassigned, chloroplast, or eukaryote were filtered. In addition, only ASVs detected in at least two samples and sequencing coverage of more than 10 were retained. ASVs of 120 samples were rarefied to 9,257 sequences per sample based on the sequencing-depth distribution and observed-feature rarefaction curves, which resulted in the exclusion of two gill samples. Potential pathogen ASVs were identified by comparison against the Multiple Bacterial Pathogen Detection database (28) and further screened for potential human pathogens using a curated database described by Littman et al. (29).

The complexity of microbial diversity was assessed through both alpha diversity and beta diversity analyses by R software (30). Alpha diversity indices were calculated using the vegan package (v2.6.6.1), and group differences were assessed using one-way analysis of variance (ANOVA) followed by Tukey’s HSD test for post hoc pairwise comparisons. Beta diversity was assessed based on weighted UniFrac distances calculated using the phyloseq package (v1.46.0) with default parameters (31). Principal coordinate analysis (PCoA) was performed based on weighted UniFrac distance matrix, and significant variations in microbiome structure across groups were tested using PERMANOVA (the adonis function in the vegan package). To identify microbial taxa with significant differential abundance between groups, linear discriminant analysis effect size (LEfSe) analysis was performed across all taxonomic levels (32). To assess the relationship between environmental factors and microbial community composition, detrended correspondence analysis (DCA) was performed to evaluate gradient length. Redundancy analysis (RDA) was applied when the length of the first DCA axis was <3.0; otherwise, canonical correspondence analysis (CCA) was conducted.

### Enumeration of foodborne pathogens by direct plating and PCR in oyster and water

The oyster tissues, including the adductor muscle, gill, gonad, intestine, and mantle (approximately 0.1 g each), were collected and placed in separate sterile 2.0 mL centrifuge tubes. Each tube contained a mixture of samples from three individuals of the same tissue type. The tissue homogenate was diluted in PBS buffer in a 10-fold gradient, with 100 μL taken from each dilution to evenly spread onto the surface of the culture medium (three replicates for each concentration gradient). The presence and densities of *Salmonella* spp. (bismuth sulfite agar), *Listeria monocytogenes* (PALCAM medium), and *Escherichia coli* (violet red bile agar) were determined by direct plating. After overnight incubation at 36°C, bacterial colonies were counted. In addition, total viable count was enumerated according to the colonies on plate count agar.

The quantification of *Vibrio parahaemolyticus* and *Vibrio vulnificus* was conducted following the method described by Tarr et al. (33) using selective media and specific virulence gene PCR methods. For *V. parahaemolyticus*, thiosulfate-citrate-bile salts-sucrose agar (TCBS) was used, the *tlh* gene was targeted (L-tlh: 5′-AAAGCGGATTATGCAGAAGCACTG-3′, R-tlh: 5′-GCTACTTTCTAGCATTTTCTCTGC-3′), which amplifies a product of approximately 450 bp. For *V. vulnificus*, peptone-sodium chloride-fiber dextrose-polymyxin E (PNCC) was used, and the *vvhA* gene was targeted, with primers as follows: Vvh-785F: 5′-CCGCGGTACAGGTTGGCGCA-3′ and Vvh-1303R: 5′-CGCCACCCATTTCGGGCC-3′, which amplify a product of approximately 519 bp.

For the detection of both pathogens, 40 characteristic colonies were selected from the TCBS and PNCC agar plates each month (if fewer than 40 colonies were present, all were tested) for PCR validation. The proportion of positive clones was calculated, representing the ratio of *V. parahaemolyticus* and *V. vulnificus* in the respective selective media for that month.

### Statistical analysis

Data on oyster biological characteristics, bacterial plate counts, and various water quality indicators were presented as mean ± standard deviation. Differences among treatment groups were evaluated using one-way ANOVA, followed by the least significant difference test for post hoc comparisons. All statistical analyses were performed using Data Processing System software. A *P* value of less than 0.05 was considered statistically significant.

## RESULTS

### Physicochemical characteristics of marine aquaculture water and biological characteristics of oysters

Beihai is located in Guangxi Province, China, while Zhanjiang is in Guangdong Province, China. The two cities are approximately 175 km apart. The sampling site in Zhanjiang is located in the inner sea region, while the sampling site in Beihai is situated in the outer sea area. The physicochemical characteristics of the seawater showed differences (Fig. S2). Water temperature ranged from 15.5°C to 30.1°C. It was similar in June and September 2023 at both locations, but the temperature in Beihai dropped to 16.33°C during winter. Water salinity in Beihai varied from 12.5 to 30 ppt throughout the year, while that in Zhanjiang was 18.5–25.6 ppt. Although pH, nitrate, and ammonium concentrations were all higher in Beihai than in Zhanjiang across all four sampling months, the exact Mann–Whitney *U* test revealed no significant differences between the two sites (*P* > 0.05). The concentrations of chlorophyll *a* ranged from 0.9 to 16.8 μg/L in Beihai and from 2.1 to 17.9 μg/L in Zhanjiang.

For the oyster samples, the shell length of *C. hongkongensis* collected in Beihai was higher than those collected in Zhanjiang (Table 1), while the oyster body weight showed no distinct difference between samples collected in December and March from both sample locations. The ratio of soft tissue in oysters collected from Beihai presented a rising trend in measured groups over time, with these values being 11.35% ± 1.89%, 13.04% ± 3.31%, 20.36$ ± 5.19%, and 22.87% ± 6.31%. A similar trend was observed in oysters collected from Zhanjiang. These findings showed that oysters from both locations exhibited an increase in soft tissue ratio over time.

### Autochthonal bacterial community in seawater

**Fig 2:**
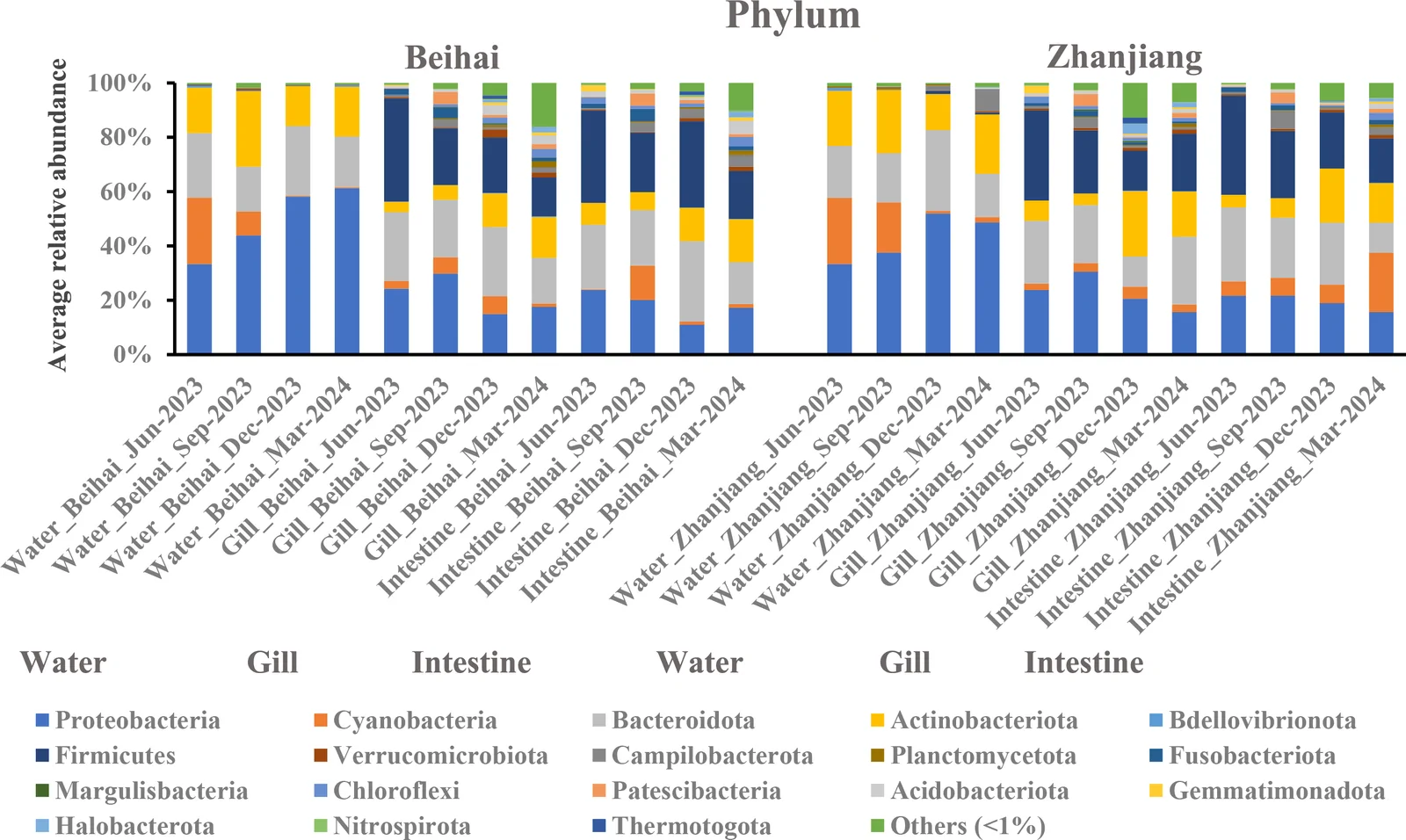
Microbial community composition at the phylum level. The average relative abundance of each taxonomic group was listed (*n* = 5 for water and intestine, *n* = 4–5 for gills).

Similar seasonal trends in water microbial community composition were demonstrated in Fig. 2 at the two harvest locations. At the phylum level, Proteobacteria, Cyanobacteria, Bacteroidota, and Actinobacteriota represented the four predominant bacterial phyla in marine water collected from two locations, Beihai and Zhanjiang. Proteobacteria was the most dominant in seawater, with a distribution ranging from 33.32% to 61.23%. Cyanobacteria were abundant in June and September 2023, which generally corresponded to warmer temperatures. Bacteroidota were shown to be stable, with a relative abundance ranging from 16% to 29.72%. The Actinobacteriota in water samples collected in December 2023 only accounted for 13.27%, while they can reach 27.72% in September 2023 (Fig. 2). At the order level, the microbial community was dominated by SAR11 (8.2%–28.41%), Flavobacteriales (13.58%–24.59%), Synechococcales (0.27%–24.4%), Rhodospirillales (2.4%–15.38%), and Microtrichales (3.65%–13.82) (Fig. S3).

### Microbial communities associated with the gill and intestine of *C. hongkongensis*

In oyster, five phyla accounted for the majority of the gill and intestine microbial community (65%–95.41%). Firmicutes and Bacteroidetes dominated (42.39%–63.85%) in most intestine microbial communities, following Proteobacteria, Actinobacteriota, and Cyanobacteria. In the gill, Firmicutes represented the numerically most abundant phylum, ranging from 14.39% to 337.98%; other predominant phyla were Proteobacteria (14.94%–30.51%) and Bacteroidota (11.11%–5.39%) (Fig. 2). Furthermore, Bacteroidales (Bacteroidetes) was the predominant order (relative abundance 6.43%–25.87%) in *C. hongkongensis*. Lachnospirales and Oscillospirales were the major members of Firmicutes in oysters. Especially, Acetobacterales belonging to Alphaproteobacteria exhibited higher abundance in June 2023 in the gill and intestinal samples (Fig. S3). Higher abundance of Synechococcales was detected in the intestine samples, reaching levels as high as 20.24%.

### Microbial diversity among different sample types

The alpha diversity indices of water microbiome were not significantly different between Beihai and Zhanjiang at each time point. The Shannon and richness indices of water communities were lower than microbial communities in oyster gill and intestine (*P* < 0.05), with minimal variation throughout the months (Fig. S4). Monthly variations revealed peaks in oyster diversity during March 2024.

**Fig 3:**
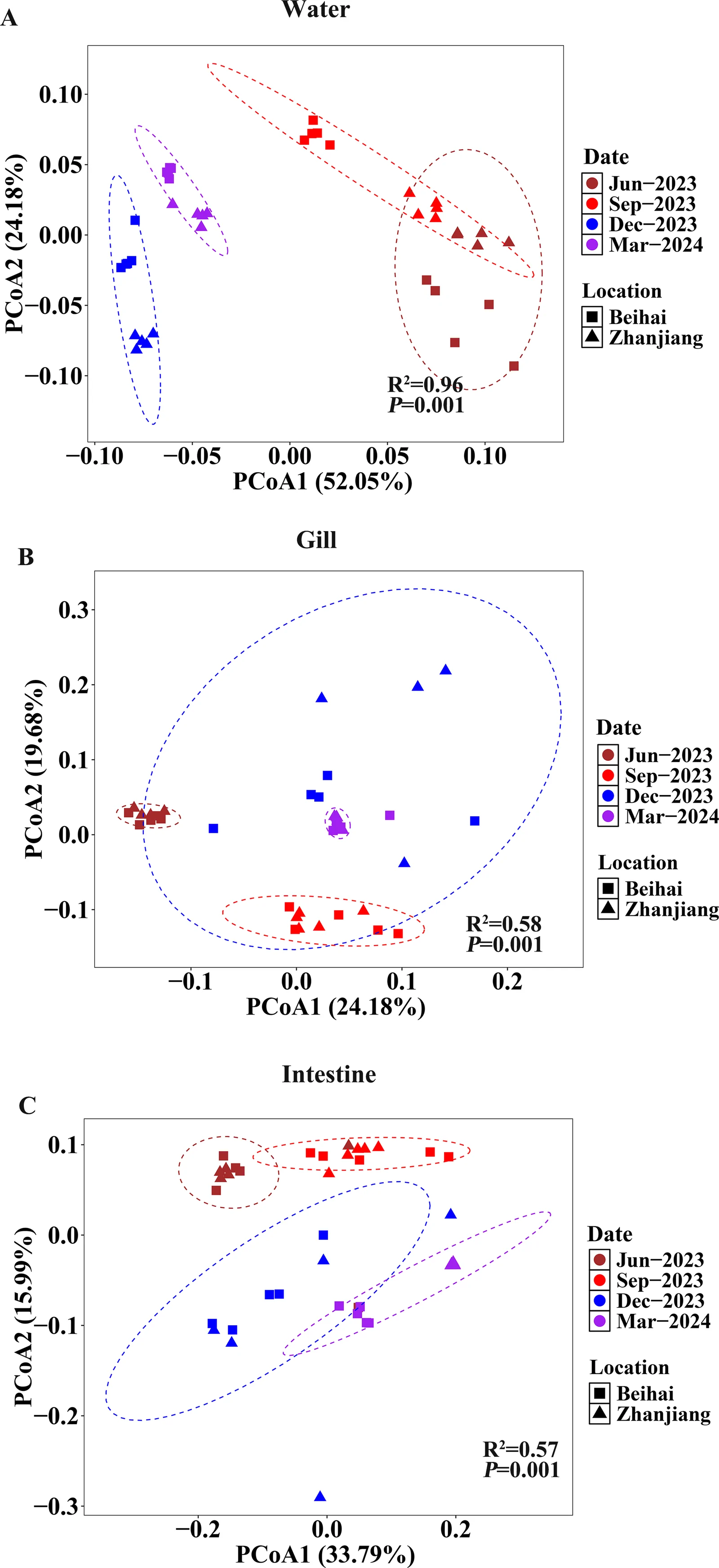
The dissimilarity between each sample type was measured using the weighted UniFrac metric. Principal coordinate analysis (PCoA) for each sample type: (**A**) water (PERMDISP, *P* = 0.002); (**B**) gill (PERMDISP, *P* = 0.013); and (**C**) intestine (PERMDISP, *P* = 0.018).

PCoA employing the weighted UniFrac metric revealed that ASVs grouped by month across each sample type. Gill samples exhibited greater similarity to intestinal samples than to water samples (pairwise PERMANOVA, *P* = 0.001) (Fig. S5). This indicated that the microbial communities in the gill and intestine were more similar to each other than to water samples. Notably, the microbial patterns of water samples exhibited marked temporal variation. Beta diversity analysis revealed substantial variability in water microbial communities across months, with apparent separation among samples from different locations within the same month. However, PERMDISP analysis indicated significant heterogeneity in multivariate dispersion among groups (PERMDISP, *P* = 0.002), suggesting that these differences may partially reflect unequal within-group variability. In contrast, oyster-associated microbial communities exhibited comparatively weaker separation between the Beihai and Zhanjiang groups (Fig. 3).

The oyster-associated microbial communities exhibited clear clustering based on the samples collected in June, September, and March, respectively (Fig. 3). Regardless of tissue type (gill or intestine), samples from different locations clustered together within the same month. Additionally, stark differences were observed in both Beihai and Zhanjiang oyster communities during December 2023. Although the gill and intestine microbial communities showed some temporal variation, they were more stable than the water community. The PERMANOVA test further revealed that the sampling month explained a large proportion of variation in water microbial communities (*R*^2^ = 0.96, *P* = 0.001), suggesting a strong temporal pattern in water microbiota, whereas fluctuations in the gill and intestine of oysters were comparatively low (*R*^2^ < 0.6, *P* = 0.001) (Fig. 3).

### Microbial community changes across time

The LEfSe analysis was used to reveal specific taxa associated with each sample during the year. For water samples, 38 bacterial clades exhibited significant differences with an LDA threshold of 4.5 across Beihai and Zhanjiang. Most of them showed significant enrichment in water from Beihai and exhibited pronounced enrichment across different sampling months. For example, at the phylum level, the relative abundance of Cyanobacteria, Actinobacteriota, and Proteobacteria significantly increased in June, September, and March, respectively. In Zhanjiang, only the water samples collected in December and March exhibited five and nine significantly enriched bacterial clades, respectively. Specifically, Rhodobacterales and Actinobacteria were the most enriched clades (with the highest LDA score) in Zhanjiang December and March water samples, respectively. (Fig. S6).

Between the two locations, oyster gill microbial communities showed fewer significantly differing taxa compared to water samples when analyzed using an LDA threshold of 4.5. Specifically, the gill microbial community in Beihai exhibited a higher abundance of Firmicutes, while the Zhanjiang gill samples showed a higher abundance of Actinobacteria. (Fig. S7). For oyster intestine samples, 16 bacterial clades showed significant differences in abundance between Beihai and Zhanjiang across time when analyzed using an LDA threshold of 4.5. The differing taxa at the phylum level included Bacteroidota, Actinobacteriota, and Cyanobacteria (Fig. S8).

**Fig 4:**
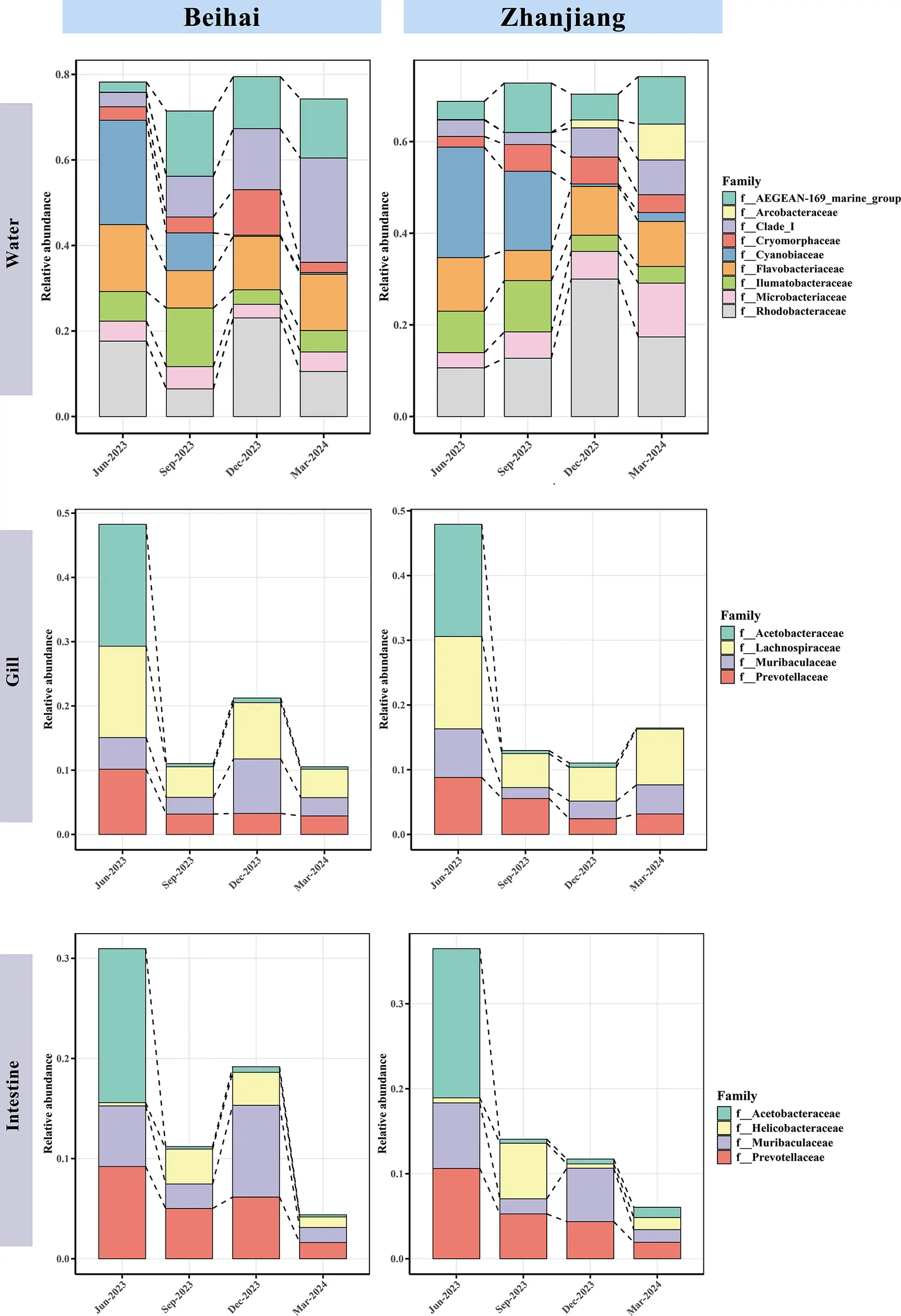
Temporal dynamics of significantly differentially abundant families across sample types in Beihai and Zhanjiang. The bacterial families shown were identified as significant biomarkers using LEfSe analysis. Taxa were selected based on a strict threshold of an LDA score of >4.5 and *P* < 0.05.

To examine the temporal dynamics of those differential bacteria, we compared their abundances across time points for the water, gill, and intestine samples. The primary differing taxa at the family level were chosen to investigate temporal dynamics (Fig. 4). Across the four time points analyzed, the water sample compositions exhibited shifts. For instance, both Cyanobiaceae and Ilumatobacteraceae showed higher abundance in June and September in both locations. Clade I (belonging to Alphaproteobacteria SAR11 clade) had a relatively higher abundance in March in both locations. Arcobacteraceae was virtually absent in the Beihai water, while it exhibited a relatively high abundance in March in Zhanjiang. Additionally, Microbacteriaceae showed obvious fluctuations in Zhanjiang water samples.

Microbial changes in gill samples from both locations were primarily associated with the families Acetobacteraceae, Lachnospiraceae, Prevotellaceae, and Muribaculaceae. Specifically, a clear trend emerged in June 2023, where Acetobacteraceae became the dominant family, accounting for most of the relative abundance. However, by September 2023, its influence had significantly decreased. For the intestine samples, they exhibited a similar level of complexity to gill samples, differing in the specific families present. Both locations showed Acetobacteraceae as the dominant family, mirroring the trend observed in the gills. Meanwhile, Muribaculaceae reached its peak abundance in December 2023 and began to decline by March 2024 in Beihai. However, its pattern differed in Zhanjiang, where a relatively high abundance was noted in June 2023 without reaching the same peak.

**Fig 5:**
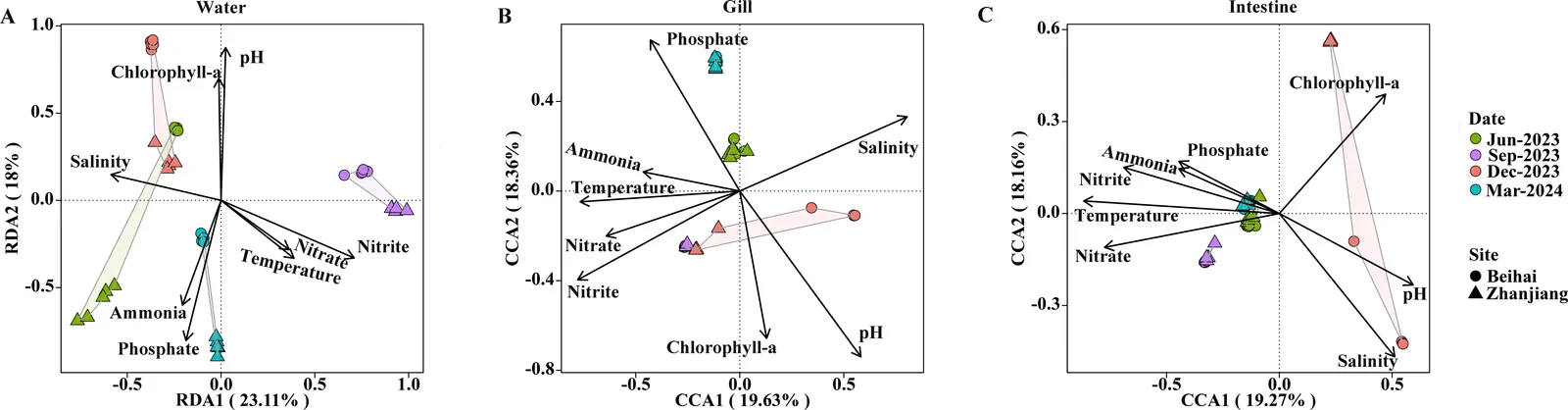
Environmental explanation of the changes in microbial community for each sample type. (**A**) RDA for significant variation of ASVs in water; (**B**) CCA for significant variation of ASVs in oyster gill; (**C**) CCA for significant variation of ASVs in oyster intestine.

To better understand the environmental factors that contributed to the changes in microbial communities, correlations among environmental factors and each sample type were analyzed (Fig. 5). The RDA was performed to explore the correlation between the water microbial community and environmental factors. As shown in Fig. 5A, 23.11% and 18% of the cumulative variance of the relationship were explained by the first and second axes of RDA, respectively. Notably, pH emerged as the key environmental factor associated with the microbial community (*R*² = 0.763, *P* = 0.001). A positive correlation was observed between the environmental factors detected in this study (temperature, nitrite, and nitrate) and the water microbial community collected in September. Water samples collected in March 2024 were mainly affected by ammonia and phosphate. For the oyster microbial community, CCA was performed. The first and second axes explained 19.63% and 18.36% of the total gill microbial variability, respectively. Among all environmental variables, pH exhibited the highest coefficient of determination (*R*² = 0.876, *P* = 0.001) in CCA for the gill-associated microbiome. The result indicated that pH was a dominant and statistically significant factor associated with the composition of the oyster gill-associated microbiome. Furthermore, the gill microbial community collected in December was affected by distinct environmental factors between the two sites: pH in Beihai and chlorophyll *a* in Zhanjiang. In the intestinal environment, temperature shows the highest *R*² (0.744, *P* = 0.001) among all measured environmental factors, indicating that it serves as a prominent factor associated with the intestinal microbial dynamics. Moreover, a similar site-specific pattern was observed in December: the intestinal microbial community was affected by chlorophyll *a* in Zhanjiang, whereas in Beihai, it was jointly influenced by salinity and pH. In addition, intestine samples collected in June and March were positively correlated with ammonia, nitrite, nitrate, phosphate, and temperature.

### Potential human pathogens inhabited in *C. hongkongensis*

**Fig 6:**
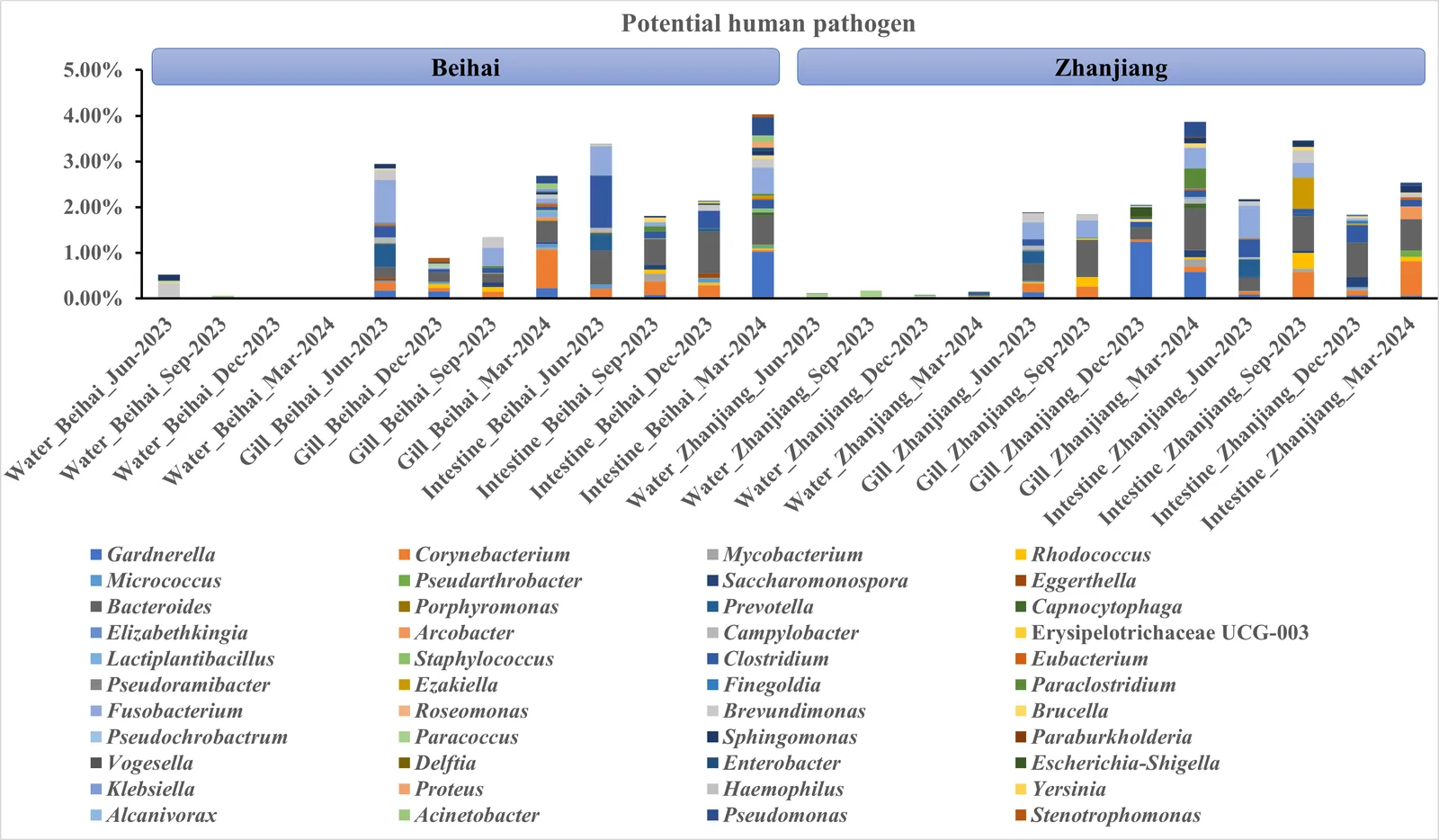
Potential human pathogens at the genus level detected by amplicon sequencing in each group (*n* = 5 for water and intestine; *n* = 4–5 for gills).

Few ASVs ascribed to potential human pathogens (It should be noted that these potential pathogen ASVs were classified solely on the basis of 16S rRNA gene sequences, which do not provide sufficient evidence to confirm the presence of virulence factors in these strains.) were detected in water of both locations. *Paracoccus* was frequently detected in Zhanjiang with a relative abundance of <0.17%, while in Beihai, the *Brevundimonas* genus was 0.32%. Oysters exhibited totally different potential pathogen ASVs from their ambient water. For oysters collected in Zhanjiang, pathogen ASVs affiliated with the genus *Gardnerella* in gills could be up to 1.24%, and pathogen ASVs associated with *Corynebacterium* were frequently detected in the intestine. For oysters in Beihai, the gill was enriched in pathogens belonging to *Fusobacterium*, *Prevotella*, and *Corynebacterium*. For intestines collected in Beihai, *Clostridium*, *Bacteroides*, *Fusobacterium*, and *Gardnerella* are the major members of the potential pathogens detected. The relative abundances of *Clostridium* and *Gardnerella* were more than 1% (Fig. 6).

### Detection of foodborne pathogens in seawater and oysters by plating method

**Fig 7:**
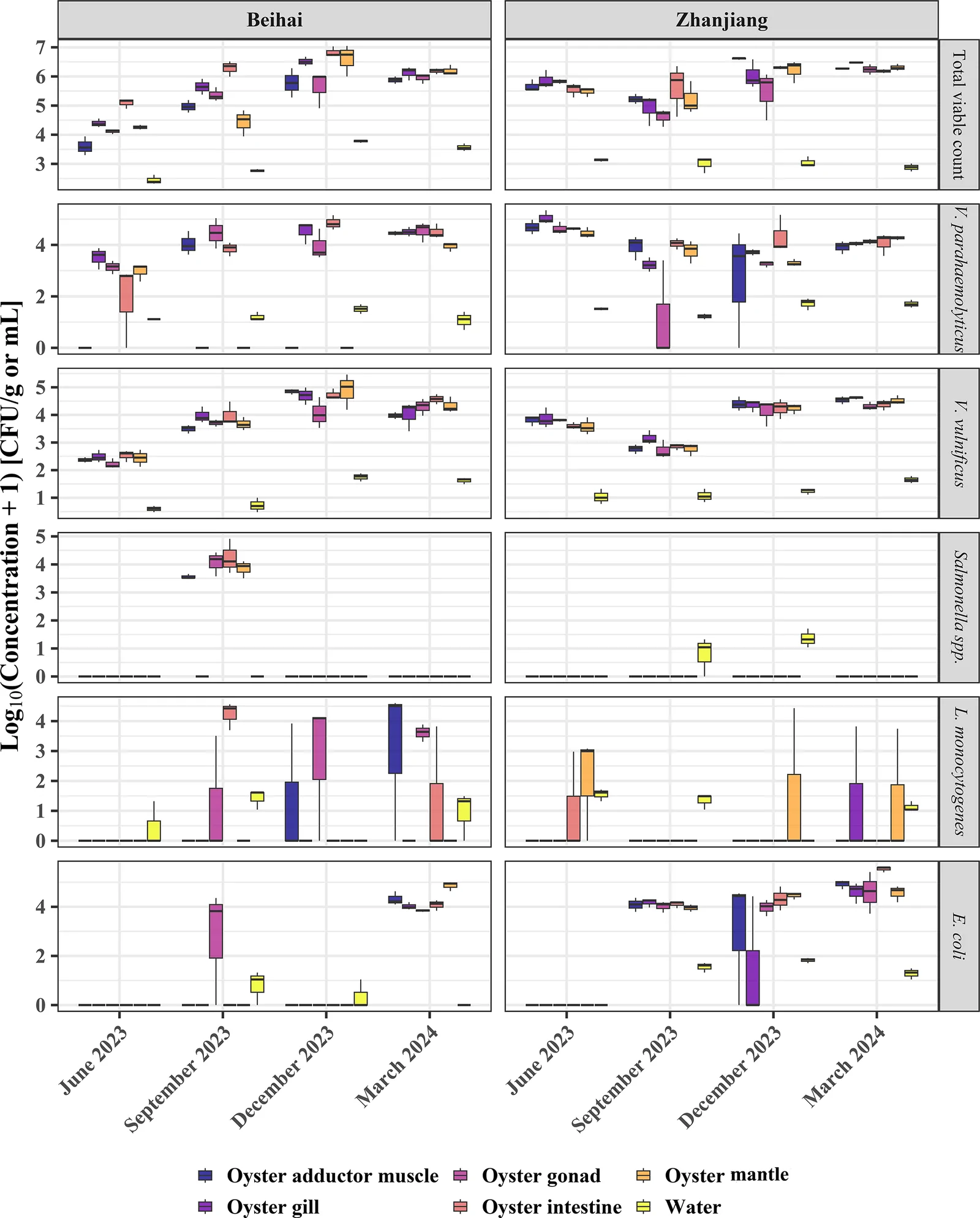
Foodborne pathogens’ concentrations in oysters and water. Each plot focuses on different microbial groups, including total viable counts, *V. parahaemolyticus*, *V. vulnificus*, *Salmonella* spp., *L. monocytogenes*, and *E. coli*, with values expressed in colony-forming units (CFU) per gram or milliliter. The samples were taken from multiple tissue types, including oyster adductor muscle, gill, gonad, intestine, mantle, and water from both Beihai and Zhanjiang, collected over four sampling dates: June 2023, September 2023, December 2023, and March 2024 (*n* = 3).

Total viable count in water samples ranged from non-detectable to 6.5 × 10^3^ CFU/mL, while in oysters, it was up to 10^8^ CFU/g. *Salmonella* spp. was detected in a limited number of samples, specifically in several oyster samples collected in September from Beihai, as well as in two water samples from Zhanjiang. In both Beihai and Zhanjiang, the *Vibrio* counts displayed seasonal fluctuations. The mean levels of *V. parahaemolyticus* and *V. vulnificus* in water from Beihai were 18.7 and 26.7 CFU/mL, while for oyster samples, the mean levels of *V. parahaemolyticus* and *V. vulnificus* were 1.87 × 10^4^ CFU/g and 2.48 × 10^4^ CFU/g. In Zhanjiang, the mean levels of *V. parahaemolyticus* and *V. vulnificus* in water were 39 and 21.3 CFU/mL, while in oysters, these levels were 2.4 × 10^4^ CFU/g and 1.5 × 10^4^ CFU/g, respectively (Fig. 7). In addition, 73.6% of all Beihai samples tested positive for *E. coli* via culture-based methods, whereas the detection rate in Zhanjiang samples was as low as 29.2%.

### Relationships between pathogens and water properties

**Fig 8:**
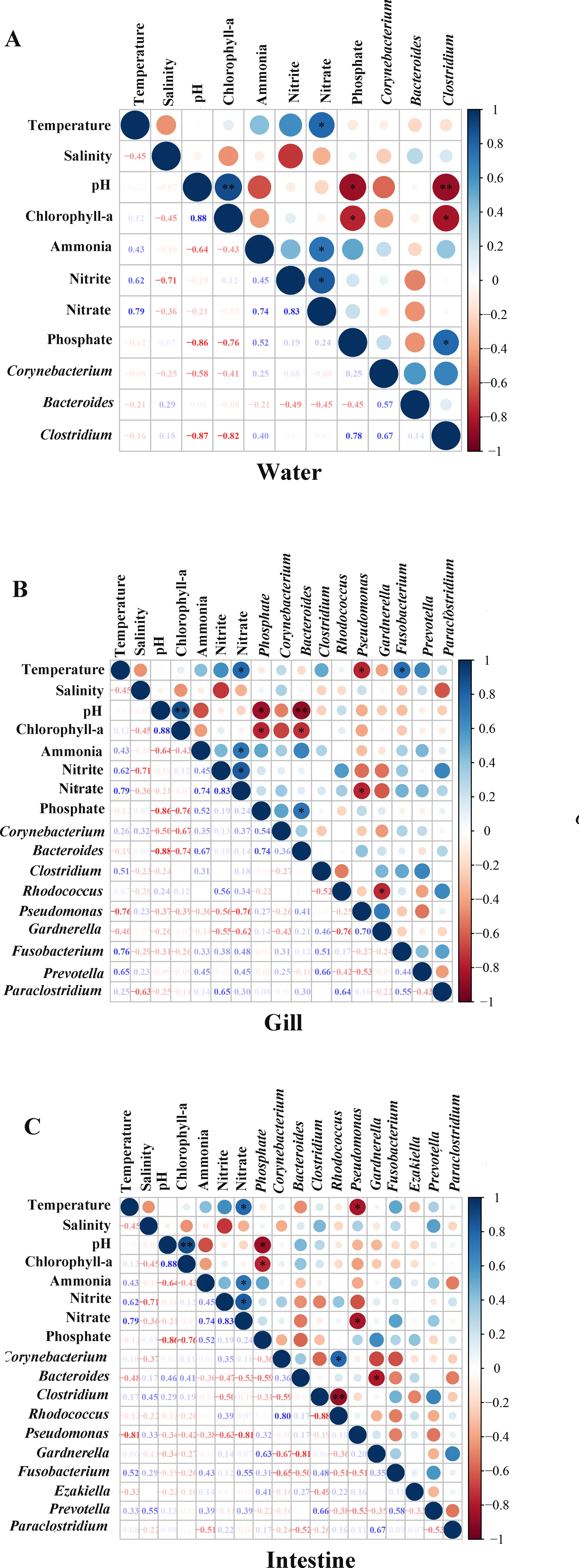
Spearman correlation analysis of the top 10 abundance-predicted pathogens at the genus level with environmental factors for water (**A**), gill (**B**), and intestine (**C**).

To clarify the associations between environmental variables and pathogens, Spearman correlation analysis was conducted between various environmental factors and pathogens, including ASV-predicted pathogens and foodborne pathogens counted by the plating method. For the predicted pathogen ASVs, fewer were found in water. *Clostridium* pathogen ASVs from water showed a significant correlation with pH and chlorophyll *a*. While for pathogen ASVs (belonging to Pseudomonas, Fusobacterium, and Bacteroides) detected in gill and intestine, they exhibited a significant relation with temperature, pH, chlorophyll *a*, and nitrate (Fig. 8).

**Fig 9:**
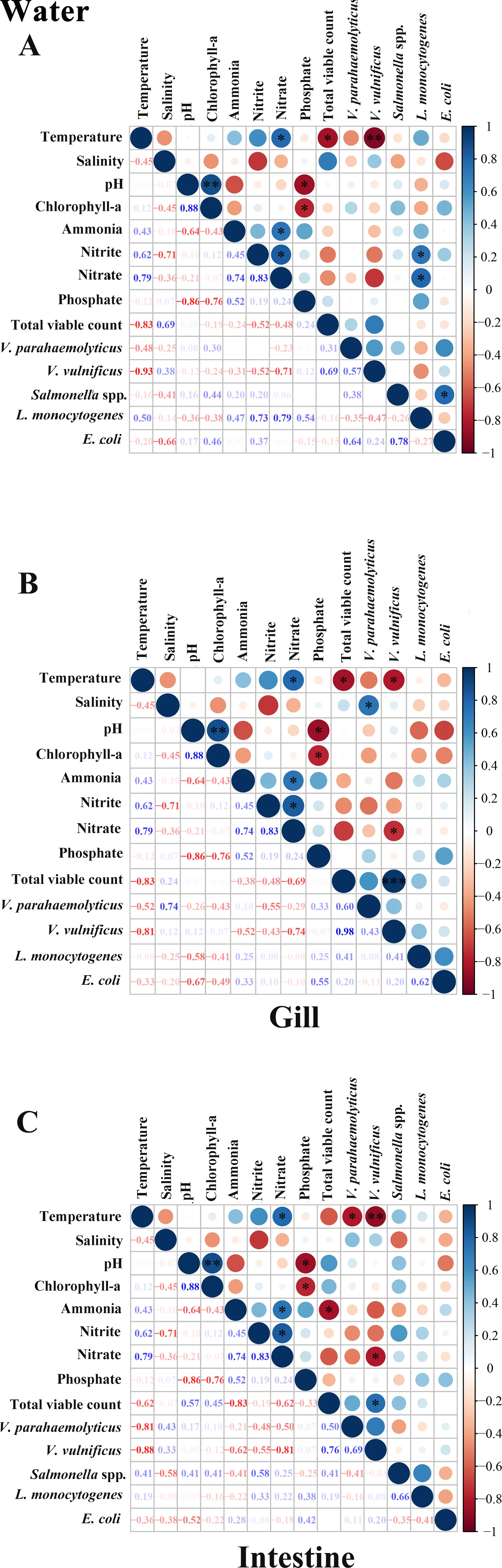
Spearman correlation analysis of enumerated pathogens with environmental factors for water (**A**), gill (**B**), and intestine (**C**).

The total viable count was negatively correlated with temperature for each sample type. In the intestine, it also showed a significant negative correlation with ammonia. The results also indicated that temperature was strongly negatively correlated with enumerated *V. vulnificus* (water: *r* = −0.93, gill: *r* = −0.81, intestine: *r* = −0.88; *P* < 0.01), suggesting that it may be an important indicator to determine the pathogen concentration. *V. parahaemolyticus* in the oyster gill was significantly positively correlated with salinity (*r* = 0.74), whereas *V. parahaemolyticus* in the intestine was negatively correlated with temperature (*r* = −0.81). *Salmonella* spp. and *E. coli* showed no significant correlation with environmental factors (Fig. 9).

## DISCUSSION

### Oyster microbiota across temporal and spatial scales

In this study, we compare the composition and annual dynamics of two geographically separated *C. hongkongensis*-associated microbial communities. The 16S rRNA amplicon data results show a significant difference between the oyster-associated microbial community and its ambient marine water, suggesting the oyster gill and intestine microbiota characterized here is mainly autochthonous. This is consistent with previous observations on the microbiota of *Crassostrea gigas* and *Saccostrea glomerata* that the host-associated microbiota is distinct from surrounding seawater planktonic microbiota (34, 35). The gut microbiota in *C. hongkongensis* feeding with *Chaetoceros muelleri* has been reported before, which illustrated that Proteobacteria were the most abundant phylum, following Firmicutes (36). In contrast, the present study showed that Firmicutes and Bacteroidetes were the most prevalent, following Proteobacteria. This may be attributed to differences in the feeding method. This suggests that environmental uptake through suspension feeding plays a significant role in shaping microbiome composition (37). Cyanobacteria (particularly Synechococcales) were abundant in the oyster microbiome. As filter feeders, oysters constantly ingest phytoplankton from seawater, and Avila-Poveda et al. (38) demonstrated that *Synechococcus elongatus* fed to oysters can systemically accumulate in their tissues. Therefore, the cyanobacterial ASVs detected in this study are likely transient members derived from ingested food, rather than true resident components of the oyster microbial community. It is also worth noting that the microbial communities in the ambient water of our study sites differed substantially from those reported in previous studies on *C. gigas* and *C. virginica*. Therefore, the distinct microbiome observed in *C. hongkongensis* may reflect not only host species specificity but also unique local environmental conditions. Further controlled experiments are needed to disentangle the relative contributions of host species and environment in shaping the *C. hongkongensis* microbiome.

Compared to water samples, the gill and intestinal microbiota were more similar to each other across most time points, though they still formed more loosely distributed clusters in the PCoA ordination, with December showing the greatest dispersion between the two tissue types (Fig. S5). The tissue-associated microbes in natural conditions exhibited a seasonal pattern and small-scale spatial variation between Beihai and Zhanjiang, indicating that host may be the major factor shaping the tissue microbiota. Similar observations have been reported in Pacific oysters (34), suggesting that their hemolymph microbial community dynamics are influenced by host population-specific factors. Host-related factors play a significant role in shaping the composition and assembly of adult oyster-gill microbial communities, as evidenced in Eastern oysters (39). In contrast, microbial populations in water ecosystems display distinct regional and seasonal variations, reflecting the complex direct interactions between environmental factors and water microbial diversity.

For oysters, little significant variation in predominant family was found across time, including Acetobacteraceae, Lachnospiraceae, Prevotellaceae, and Muribaculaceae. This suggests that these bacteria are the major members of the oyster-associated microbiome, and they are resilient to the changing environment. Acetobacteraceae are typically found in flowers, paddy soils, sludge, and the guts of insects, and some of the strains may supply the host with amino acids (40, 41). Members of the family Lachnospiraceae colonize diverse ecosystems. They are predominantly found in the gastrointestinal tracts of multiple hosts, including humans, mice, and insects. Additionally, certain species are enriched in anaerobic soil, aquatic sediments, and hydrothermal vents (42). It has been reported that Lachnospiraceae plays important roles in human health and disease. For example, some Lachnospiraceae could produce short‐chain fatty acids and vitamins that may have effects on anti‐inflammatory, immunity‐inducing, and homeostasis-maintaining processes (43). In addition, certain members of Lachnospiraceae are characterized as probiotics to inhibit enteropathogens (44). Prevotellaceae has also proven to be the most abundant bacterial family in the healthy human gut, some lineages of Prevotellaceae possess sialidases, indicating that they have important functions impacting host health (45). Moreover, Muribaculaceae, as the primary commensal bacteria in the gut microbiota, belong to the order Bacteroidetes and are known for producing short-chain fatty acids, making them a potential probiotic bacterial family (46).

### Correlations between environmental parameters and oyster microbial communities

In the present study, oysters from the same *C. hongkongensis* family were selected from two locations to minimize genetic influences as a confounding factor. This design allowed us to investigate how environmental characteristics shape the oyster microbiota. For the gill tissue, the associated microbiome was not significantly different between two locations, except for a change in the distance decay relationship in community composition in December. In gill samples collected in December, environmental differences were observed between Beihai and Zhanjiang. Concurrently, the Beihai area was characterized by elevated salinity and pH values, whereas the Zhanjiang area displayed significantly higher concentrations of nitrite, nitrate, and chlorophyll *a*. Concurrently, differences in microbial community composition were detected between the two locations. ASVs belonging to Muribaculaceae were more abundant in Beihai, whereas members of Actinobacteria and Coriobacteriia (phylum Actinobacteriota) were more prevalent in Zhanjiang. These observed variations in microbial communities may be related to the differences in environmental factors (e.g., pH, salinity, and nutrient levels), though further validation is needed to confirm potential causal relationships. Notably, the environmental characteristics of Zhanjiang, specifically its location in the inner sea, may provide context for the higher nitrite, nitrate, and chlorophyll *a* concentrations observed in its December samples, as inner sea environments often have relatively limited water exchange, which can facilitate nutrient accumulation compared to more open coastal areas like Beihai. This potential nutrient-rich condition in Zhanjiang aligns with the higher prevalence of Actinobacteriota (encompassing Actinobacteria and Coriobacteriia) observed there, further supporting the notion that microbial community variations between the two sites could be linked to environmental gradients (e.g., nutrient availability). However, as with the broader environmental–microbial associations noted earlier, direct causal links between nutrient levels and Actinobacteriota abundance would require additional evidence, such as statistical correlations or functional studies. For intestine samples, seasonal changes in environmental factors may be primarily driven by temperature, consistent with previous studies that have characterized the influence of temperature on the oyster-associated microbiomes (11, 22, 35). Specifically, in December, chlorophyll *a*, salinity, and pH emerged as the major factors potentially affecting the intestine-associated microbiome. Notably, environmental factors exerted similar effects on intestine and gill microbiota. However, spatial differences in the intestine-associated microbial communities between Beihai and Zhanjiang were predominantly associated with variations in the relative abundance of Bacteroidia and *Marivita* (Fig. S8). Bacteroidia are primarily anaerobic members and are well-known in the guts of humans and animals (47). It has been reported that *Bacteroides* metabolize organic matters, potentially providing nutrition and vitamins to the host and other intestinal microbial residents (48). *Marivita*, a genus within the Rhodobacteraceae family, has been reported to promote growth under high nitrate conditions (49). These fine-scale differences between the two locations further illustrate the potential acclimation capacity of both gill and intestinal microbiomes.

### Correlations between environmental parameters and potential pathogens

Based on the amplicon sequencing data, most human potential pathogens predicted in oysters were virtually absent from the ambient seawater microbial communities. However, it remains unclear whether these pathogens are resident or transient members of the oyster microbial community. Additionally, foodborne pathogens detected via plate counting were not identified in the ASV data sets. This discrepancy between plating enumeration and amplicon-based abundance is most likely attributable to methodological biases (such as primer bias). Therefore, integrating both culture-dependent and culture-independent approaches provides a more comprehensive assessment of the microbial risk profile. Specifically, our analysis then focused on whether annual seasonal environmental fluctuations (e.g., in temperature, nutrient levels, or salinity) are sufficient to trigger an increase in opportunistic pathogens across different sample types.

Across both sampling locations and throughout the year, oyster samples consistently contained a greater number of pathogen-associated ASV types compared to seawater samples. Previous studies have revealed that factors leading to the accumulation of pathogens in oysters corresponded with warmer temperatures, lower salinity, and cyanobacteria (6). In our analysis of pathogen-associated ASVs in *C. hongkongensis*, we observed more nuanced relationships with temperature. *Pseudomonas* exhibited a significant negative correlation with temperature, while *Fusobacterium* showed a significant positive correlation. Other detected pathogens, by contrast, had no significant association with temperature. These divergent responses highlight the complexity of individual pathogen taxa’s environmental preferences within the oyster host.

For foodborne pathogens detected via plate counting, *Vibrio* was the most frequently identified genus, consistently detected in oyster tissues, with a lower detection rate in seawater samples. It has been reported that *V. vulnificus* and *V. parahaemolyticus* were susceptible to temperature, salinity, and chlorophyll *a* (50, 51). However, our study observed no significant correlations between chlorophyll *a* and total *Vibrio* concentrations, nor between salinity and total *Vibrio* concentrations, with one exception: *V. parahaemolyticus* showed a significant positive correlation with salinity in gill samples. Thus, it is possible that pathogenic *V. parahaemolyticus* responds differently to salinity in different sample types. Furthermore, it has been recognized that a non-linear relationship cannot be reliably identified when salinities do not exhibit a sufficiently wide range of variation or when such variation is confined to one side of the optimal salinity level (50). In addition, few studies have reported that elevated temperatures could increase the prevalence of *Vibrio* in the microbiome of oysters (52) and scallops (53). However, temperature was negatively correlated with *Vibrio* here.

### Conclusion

To address critical knowledge gaps regarding the seasonal dynamics of *C. hongkongensis*-associated microbiomes, quantify potential bioaccumulation risks of pathogens in oyster tissues, and identify key environmental drivers that may shape microbial communities, we conducted a year-long longitudinal sampling campaign. This study provides preliminary evidence suggesting that the structural and functional attributes of oyster-associated microbiota may play a pivotal role in mediating host disease resistance, potentially advancing our understanding of microbial community dynamics within complex natural coastal ecosystems. Furthermore, these findings may lay a foundational framework for the future development and optimization of microbiota-based biotherapeutic strategies, offering actionable insights that could enhance disease management and sustainability in global oyster aquaculture.

## Data Availability

The sequencing data generated in this study have been deposited with NCBI (BioProject PRJNA1494816) (Table S1).

## SUPPLEMENTAL MATERIAL

Supplemental figures aem.01056-26-s0001.docx Fig. S1 to S8.

Table S1 aem.01056-26-s0002.xlsx Sequencing sample metadata and NCBI accessions.

ASM does not own the copyrights to Supplemental Material that may be linked to, or accessed through, an article. The authors have granted ASM a non-exclusive, world-wide license to publish the Supplemental Material files. Please contact the corresponding author directly for reuse.
