# Supplementary material for: Seasonal dynamics of microbial communities and potential human pathogens in Crassostrea hongkongensis and ambient water in southern China
Source: Appl Environ Microbiol. 2026 Sep 2;92(9):e01056-26. doi: 10.1128/aem.01056-26 (PMC13599550; doi:10.1128/aem.01056-26)
Supplement: Supplementary file 1 [file aem.01056-26-s0001.docx]

**Figure S1. Schematic diagram of the experimental workflow for oyster.**

**Figure S2.** The varying physicochemical characteristics of seawater samples (*n* = 3) during June 2023 to March 2024. (A) Temperature; (B) pH; (C) Chlorophyll-a; (D) Salinity; (E) Ammonia (NH_4_^+^-N); (F) Nitrite (NO_2_^−^-N); (G) Nitrate (NO_3_^−^-N); (H) Phosphate (PO_4_^3−^).

**Figure S3.** Microbial community composition at the order level. The average relative abundance of each taxonomic group was listed. (A) microbial composition in water (*n* = 5); (B) Microbial composition in oyster (*n* = 5 for intestine; *n* = 4–5 for gill).

**Figure S4.** Alpha diversity analyzed by sample type and month.

**Figure S5.** Principal coordinates analysis (PCoA) for sample types in each month. (A) Jun-2023 (PERMDISP, *P*=0.058); (B) Sep-2023 (PERMDISP, *P*=0.001); (C) Dec-2023 (PERMDISP, *P*=0.003); (D) Mar-2024 (PERMDISP, *P*=0.152).

  **Figure S6.** [LEfSe](https://www.sciencedirect.com/topics/agricultural-and-biological-sciences/discriminant-analysis) analysis of water microbial abundance in Beihai and Zhanjiang across the year.

**Figure S7.** [LEfSe](https://www.sciencedirect.com/topics/agricultural-and-biological-sciences/discriminant-analysis) analysis of water microbial abundance in oyster gill collected from Beihai and Zhanjiang across the year.

**Figure S8.** [LEfSe](https://www.sciencedirect.com/topics/agricultural-and-biological-sciences/discriminant-analysis) analysis of water microbial abundance in oyster intestine collected from Beihai and Zhanjiang across the year.
